# Intra-articular steroid injections: 5 years' experience in a tertiary paediatric rheumatology centre

**DOI:** 10.1186/1546-0096-12-S1-P146

**Published:** 2014-09-17

**Authors:** Rebecca A James, Jane Munro, Roger Allen, Angela Cox, Peter Gowdie, Jo Buckle, Jonathan Akikusa

**Affiliations:** 1 Rheumatology, Royal Children's Hospital, Melbourne, Australia

## Introduction

Intra-articular steroid (IAS) injections are a safe and effective therapy in the management of Juvenile Idiopathic Arthritis (JIA). Timely access to joint injections, including appropriate anaesthesia, is now considered standard of care in the treatment of JIA. Despite this, little is known about the resource burden created by IAS provision. Published data is also lacking to inform families of the likely role that IAS will play in the management of their child’s disease.

## Objectives

To describe the role of intra-articular steroid injections in the management of a large cohort of patients with Juvenile Idiopathic Arthritis at a tertiary paediatric Rheumatology centre over a 5 year period, as well as the resource burden this created.

## Methods

All patients with Juvenile Idiopathic Arthritis who attended the Royal Children’s Hospital, Melbourne, over a 5 year period between 1 January 2009 and 31 December 2013 were identified using our computerised database. This is a comprehensive clinical tool coded in Microsoft Access, which is used to record all Rheumatology patient demographics, contacts, procedures, communications and therapies. Data were extracted into an Excel spreadsheet and analysed using descriptive statistics. For the purpose of this study, multiple joint injections performed on the same patient on the same day were considered to be a single joint injection procedure.

## Results

A total of 544 patients with JIA were cared for during the study period, including 337 patients with a new diagnosis of JIA. 338 (62.0%) of these patients were female. 410 patients (75.4%) underwent 892 joint injection procedures during the study period. 419 of these procedures (47%) were performed under general anaesthetic, with a median patient age of 6.48 years (range 1.12-18.53). 473 procedures (53.0%) were performed under nitrous oxide sedation, with a median patient age of 12.66 years (range 1.71-20.00). Of newly diagnosed patients who required joint injection, median time to first IAS was 35 days (range 0-1169). 87% of new patients who required joint injections during follow up were injected within 6 months of diagnosis. Throughout the study period, new JIA diagnoses increased by 20.90%, joint injections procedures increased by 7% and approvals for biologic agents increased by 200%.

## Conclusion

Intra-articular steroid injections are a core aspect of the management of patients with JIA, with over 75% of patients requiring injection over a five year period. General anaesthetic was required in nearly half (47%) of these procedures, and was most important in younger patients. Of newly diagnosed patients who required IAS injections, nearly 90% were injected within 6 months of diagnosis.

## Disclosure of interest

None declared.

